# A Method for Preparing Diamond Films with High Thermal Stability

**DOI:** 10.3390/nano15211606

**Published:** 2025-10-22

**Authors:** Xia Zhao, Chao Han, Xin Jia, Zifeng Fan

**Affiliations:** 1 Beijing Institute of Metrology, Beijing 100029, China; 2 National Institute of Metrology, Beijing 100029, China; 3 Beijing Institute of Standardization, Beijing 100013, China

**Keywords:** diamond films, thermal stability, preparation method, wide application

## Abstract

Due to the outstanding thermal stability of diamond film, diamond films have extensive application prospects in fields such as electronics, optics, biomedicine, and aerospace, and are one of the important materials driving the development of modern science and technology. Moreover, the cost of single-crystal diamond substrates is high, and it is difficult to achieve large-scale batch production. A direct current arc plasma jet chemical vapor deposition method, combined with post-treatment steps such as nano-diamond seed crystal implantation, surface modification, and high-temperature annealing, is used to prepare high-quality diamond films. The relationship between the thermal conductivity and optical properties of diamond films is analyzed in detail. The experimental results showed that diamond film has a relatively smooth surface, with a surface roughness that can reach 3 nm. As the temperature rises, diamond films exhibit good crystal orientation and thermal stability, the FWHM of reflection peaks become smaller, and thermal conductivity can reach 1734 W/(m·K). The infrared testing analysis also confirmed that the diamond film has excellent thermal diffusion properties. When the diamond film is applied to power device chips, it can effectively reduce the junction temperature of 30 °C. The preparation method proposed in this paper is expected to break through the cost and scale limitations of high-performance diamond films, thereby promoting the wide application of diamond films in industries.

## 1. Introduction

Diamond is an excellent material for electronic and photonic applications due to its outstanding carrier mobility, thermal conductivity, dielectric breakdown strength, ultra-wide bandgap, and optical transparency [1,2]. CVD diamond also exhibits extremely high mechanical strength and thermal conductivity (up to 2000 W/(m·K)), enabling it to resist thermal stress and deformation caused by supersonic thermal shock, thereby reducing optical distortion [3,4,5]. These characteristics make diamond films the ideal choice for long-wave infrared windows in supersonic aircraft [6,7]. Professor Yu Dafang from Xiamen University prepared single-crystal diamond. When the diamond was reduced to a thickness of several tens of nanometers, it still maintained excellent in-plane thermal conductivity (approximately 2000 W/(m·K)) [8]. Professor Bing-Yang Cao conducted a comparison between CVD diamond films grown on Si substrates and those on 3C-SiC/Si substrates. He discovered that increasing the grain size of the diamond films and reducing the content of amorphous carbon would help enhance their thermal conductivity [9]. The Cheng-Yun Wang team [10] used a scanning thermal microscope (SThM) to create a nanoscale thermal conductivity distribution map, conducting research to evaluate the quality of grain boundaries and the disorder of the microstructure on the thermal properties of polycrystalline diamond films. M. Fischer’s team has developed a technology for depositing ultra-thin polycrystalline diamond films on silicon wafers. This technology can reduce the thermal load of electronic components by ten times and increase the charging speed of electric vehicles by five times [11]. Professor Xiong Ying [12,13,14,15] utilized the extremely high thermal conductivity of diamond and the flexibility and high specific surface area of graphene to achieve a comprehensive performance of high thermal conductivity, low thermal resistance, and good compressibility. The L Yang team [16,17] has solved the serious warping problem of diamond films that occurs after their growth due to residual stress.

In general, a positive correlation exists between the thermal conductivity and infrared transmittance of CVD diamond films [18]. However, from a microscopic perspective, the optical quality is primarily determined by the material’s photon absorption characteristics. These characteristics are influenced by multiple factors, including microstructure, surface roughness, free carrier absorption, thickness, purity, density, grain size, and impurities [19,20]. In contrast, the thermal properties are governed by the mean free path of phonons, which is predominantly limited by grain boundaries, chemical impurities, dislocations, and vacancies [21]. Consequently, because the underlying influencing factors for optical and thermal performance are distinct, their relationship cannot be described by a simple linear model. Given the challenge in fabricating idealized CVD diamond, identifying the correlation between these properties and analyzing the key factors is crucial for advancing the practical application of CVD diamond window materials. Therefore, the synthesis of CVD diamond that combines high optical quality with high thermal quality is crucial for realizing its application in optical windows. Although researchers have conducted extensive studies on the thermal conductivity of diamond films, there has been little research on the thermal stability of diamond films at high temperatures, as well as the interrelationship between their thermal conductivity and optical properties.

To address the above issues, this paper is based on the direct current arc plasma jet CVD method, combined with the post-treatment of nano-diamond seed crystal implantation, surface modification, and high-temperature annealing to prepare a process for high-quality diamond films. The thermal stability of diamond films at high temperatures, as well as the interrelationship between their thermal conductivity and optical properties, were also analyzed. Finally, the excellent heat dissipation effect of the diamond film is verified through the power device chip. The research on the thermal properties of diamond films is not only crucial for solving the current problem of heat dissipation in electronic devices, but also has far-reaching significance for the technological upgrading and innovation in the fields of semiconductors, aerospace, and energy.

## 2. Method

### 2.1. Preparation of Diamond Film

Polycrystalline CVD diamond films are fabricated using a 100 kW class direct current arc plasma jet chemical vapor deposition system with titanium-coated graphite as the substrate (Table 1). The specific operational steps are as follows:

(1) Polish the Si substrate with a silica sol solution (100 nm particle size, 40% concentration) for at least 30 min to remove the surface oxide layer.
(2) Subject the Si substrate to ultrasonic treatment in an acetone suspension containing diamond micropowder. The suspension is prepared using diamond micropowder with a particle size ranging from 0.2 to 1 μm at a concentration of 3–6 g per 100 mL of acetone. The seeding process lasts 30–60 min. Subsequently, clean the substrate ultrasonically in anhydrous ethanol for 10–15 min. Finally, dry the substrate surface using compressed nitrogen for further use.
(3) Modify the surface of the prepared nitrogen-doped micron diamond film using reactive ion etching (RIE) technology. Then, clean it ultrasonically in anhydrous ethanol for 5–10 min.
(4) The obtained samples are placed in a muffle furnace for high-temperature vacuum annealing treatment. The vacuum pressure is maintained at 10^−4^ pa for a duration of 2 h to obtain a high-strength diamond film.

### 2.2. Characterization of Diamond Film

The thermal conductivity of the diamond film was measured using an NETZSCH LFA467 laser flash analyzer (NETZSCH, Selb, Germany). Thermal diffusivity and thermal conductivity were determined via the laser flash method. For each group, three samples were tested, and the average value was calculated. The optical properties of the diamond film within the mid-infrared range (400–4000 cm^−1^) were analyzed using a Fourier transform infrared spectrometer (Excalibur 3100, Varian Inc., Palo Alto, CA, USA) with a resolution of 4 cm^−1^. The crystal quality was examined by confocal Raman spectroscopy (Via-Reflex system, B&W Tek, Shanghai, China). Surface morphology, grain size, and grain boundary distribution were characterized using a high-depth 3D optical microscope (VHX-6000, KEYENCE Corporation, Osaka, Japan).

### 2.3. The Calculation Method of Thermal Conductivity

The thermal conductivity model of diamond films can be expressed by the following formula:

κ = α · ρ ·C_p_ (1)

where ρ is density, α represents the thermal diffusivity coefficient, and C_p_ is specific heat capacity.

The density and specific heat capacity of diamond in this study are taken as follows: ρ ≈ 3.5 g/cm^3^, Cp ≈ 0.5 J/g·K. The thermal diffusivity (α) was directly measured by the instrument LFA467 laser flash analyzer (NETZSCH, Selb, Germany). The diamond film sample was measured three times, and the average value was taken as the final measured value of the thermal diffusivity. Because the diamond film is a self-supporting film, the interface thermal resistance was ignored in this study. Since diamond films have low optical absorption, a metallic transducer layer of gold was coated on the film surface to enhance laser energy absorption, ensuring reliable temperature detection during the measurement. According to the above measurement process, the thermal conductivity of the diamond film can be obtained.

## 3. Results

### 3.1. The Morphological Characteristics of Diamond Film

**Figure 1:**
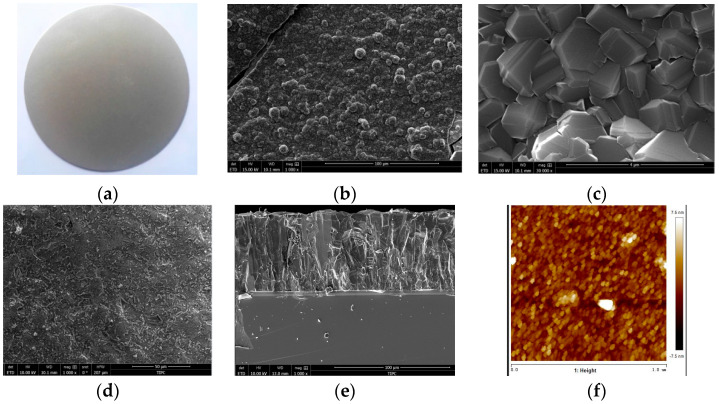
SEM morphology characterization and surface roughness of diamond film. (**a**) Diamond film; (**b**) surface morphology; (**c**) crystal structure; (**d**) surface morphology; (**e**) cross-sectional morphology; and (**f**) surface roughness.

Shown in Figure 1a is diamond film with a diameter greater than 120 mm. It is a diamond film after grinding treatment. After 10 h of epitaxial growth, a dense crystal diamond continuous film is formed, as shown in Figure 1b. Figure 1c shows an enlarged view of the diamond crystal structure. As the epitaxial growth time increases, the surface of the hexagonal structure gradually closes into a continuous and smooth film. After 15 h of epitaxial growth, a flat polycrystalline diamond film surface is formed, as shown in Figure 1d. The results show that the diamond film has the characteristics of a complete crystal structure and very few defects. It can achieve large-area and uniform epitaxial growth and has a smooth surface. Figure 1e shows the cross-sectional scanning electron microscope image of the diamond film. It can be observed that the film has a columnar crystal structure and a thickness of 70 μm. The interface between the film and the substrate is clear, indicating that the diamond film has good compactness and adhesion.

Figure 1f shows the surface roughness of diamond film. To verify the surface smoothness of the diamond film, we use the atomic force microscope (Asylum Research, Santa Barbara, CA, USA) to measure the surface roughness of the diamond film. We select 3–5 different representative areas on a sample for scanning to obtain statistically significant roughness data, avoiding measurement at only one point. Figure 1f shows that the surface roughness of diamond reaches 3 nm, which is sufficient to prove its smooth surface.

### 3.2. The XRD Patterns of Diamond Film

**Figure 2:**
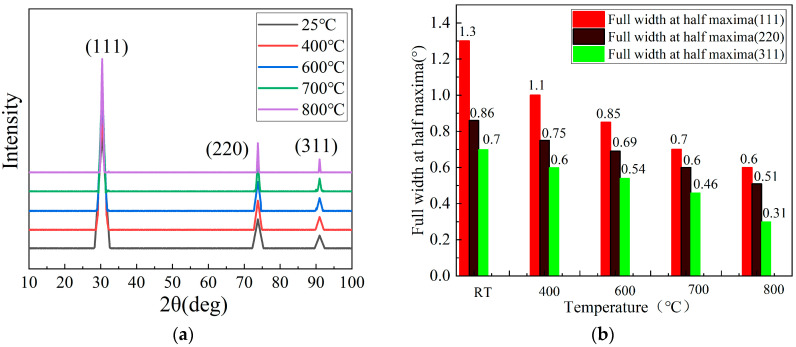
(**a**) XRD patterns of diamond film heated at different temperatures. (**b**) The FWHM of reflection peaks.

Figure 2 shows the XRD patterns of diamond film heated at different temperatures, as can be seen from Figure 2a, at room temperature of 25 °C, the centers of the diffraction peaks of 111, 220 and 311 appear at 29.79°, 74.98° and 92.24°, respectively. When the heating temperature rises from 400 °C to 800 °C, the positions of the diffraction peaks do not change significantly and still remains in original position, without any new diffraction peaks. The diffraction peaks at 29.79°, 74.98°, and 92.24° become more prominent with the increase in temperature. These indicate that the thermal stability of the diamond film is very good and it can crystallize better at high temperatures.

Figure 2b shows the full width at the half maxima of diffraction peaks of 111, 220, and 311 for diamond film. As the temperature increases, the reflection peaks (111, 220, and 311) gradually decrease. The FWHM of reflection peaks (111) decreased from 1.3 to 0.6, with the largest decline. The FWHM of reflection peaks (220 and 311) decreased from 1.3 to 0.51 and 0.7 to 0.31, respectively, which is more conducive to the formation of the crystal structure of the diamond film.

**Figure 3:**
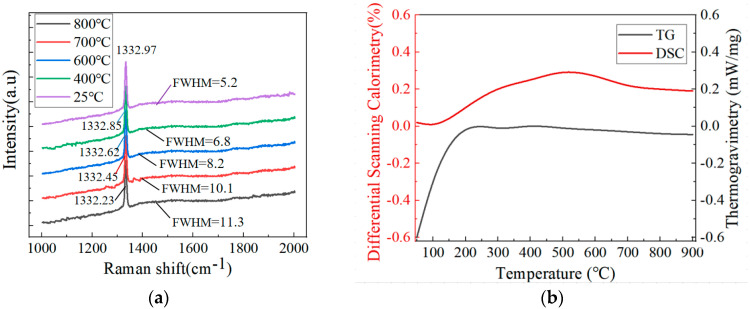
(**a**) Raman spectrum of diamond film heated at different temperatures, (**b**) DSC-TG curves of diamond film.

Figure 3a shows the Raman spectrum of diamond film heated at different temperatures. From Figure 3a, peak D of the diamond film at different temperatures occurs around 1332 cm^−1^, and the half-height width is more than 5 cm^−1^. As the temperature rises from 25 °C to 800 °C, the position of the D peak of the diamond film decreases, changing from 1332.97 cm^−1^ to 1332.23 cm^−1^, and the FWHM gradually increases, changing from 5.2 cm^−1^ to 11.3 cm^−1^. The position and FWHM of peak D do not show significant changes with temperature, and no other peaks are found at other locations. It indicates that the crystal structure of diamond films is relatively stable and can maintain a good crystal structure even at high temperatures.

In Figure 3b, the TG curve indicates that during the heating process, there is no significant weight loss change to the diamond films prepared by the direct current arc plasma jet CVD technology, remaining at 0.1 mW/mg. The DSC curve is relatively smooth, without obvious changes or heat absorption and release peaks, suggesting that the diamond film has good thermal stability and does not undergo oxidation or decomposition at high temperatures.

### 3.3. The Thermal Conductivity and Optical Properties of Diamond Film

**Figure 4:**
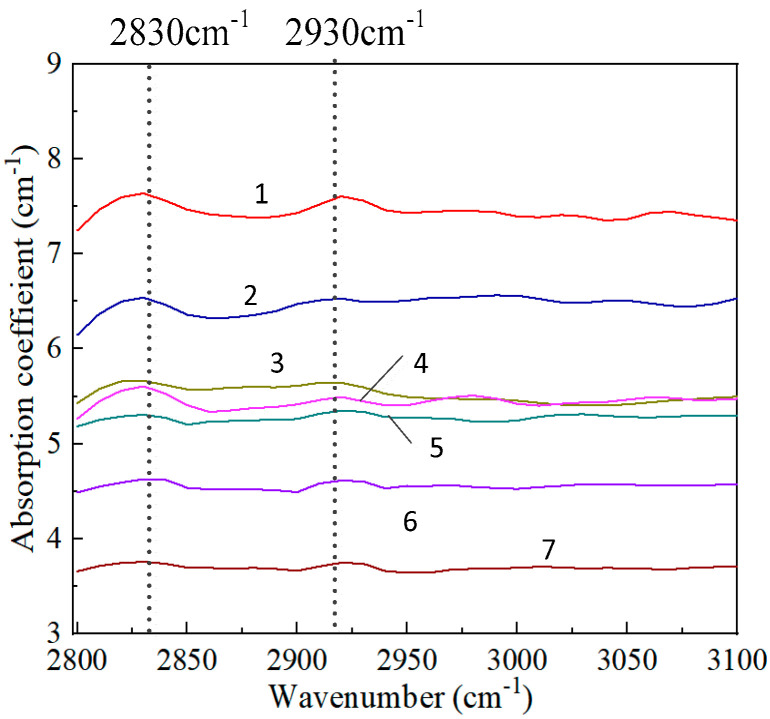
The absorption coefficient of the C-H stretching vibration in the 3.30–3.60 μm wavelength range.

Figure 4 shows that in the infrared spectrum at the wavelength range of 3.3–3.6 μm (2780–3030 cm^−1^), there is a C-H vibration absorption [22]. There are two distinct absorption peaks, located at 2830 cm^−1^ and 2930 cm^−1^, respectively. This is because the absorption peak near 2830 cm^−1^ is related to the hydrogen being restricted in the defects within the crystal, while the absorption peak near 2930 cm^−1^ is associated with sp^2^ carbon [23]. The presence of these hydrogen impurities and sp^2^ carbon both have adverse effects on the thermal conductivity and infrared transmittance of the diamond film.

**Figure 5:**
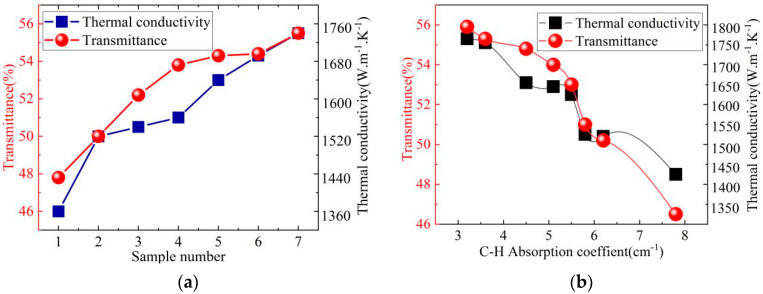
(**a**) Thermal conductivity and transmittance. (**b**) Thermal conductivity and average infrared transmittance change with absorption coefficient (2830 cm^−1^).

Figure 5a shows the thermal conductivity and average transmittance of seven diamond film samples. Figure 5a indicates that the average transmittance is basically positively correlated with the thermal conductivity, and both the thermal conductivity and infrared transmittance show a rapid increase and gradually moderate process. The range of thermal conductivity is from the lowest 1387 W/(m·K) to 1734 W/(m·K). The transmittance range of diamond films at 8 to 12 μm is 47.78% to 55.36%.

Figure 5b shows the variation laws of thermal conductivity, transmittance and absorption rate of diamond films near the absorption peak (2835 cm^−1^). The absorption coefficient at 2834 cm^−1^ varies inversely with the transmittance and thermal conductivity, and there is a good linear relationship between the thermal conductivity and the absorption coefficient. This is mainly due to the certain correlation between the C-H bond and non-diamond carbon. When the absorption coefficient of the C-H bond is relatively high, the infrared transmittance shows a good linear relationship with the absorption rate of the C-H bond. However, when the absorption coefficient of the C-H bond decreases to 5.5 cm^−1^, the correlation between the absorption coefficient of the C-H bond and the infrared transmittance weakens.

### 3.4. Analysis of the Heat Dissipation Performance of Diamond Film

To visually verify the high thermal conductivity of diamond films and their effectiveness in heat dissipation applications, compare the surface temperature field distribution of the diamond film samples and the Si film samples during the heating process to 200 °C. Before measurement, all sample surfaces must be cleaned to ensure there is no dust. Turn on the thermal imager and record the temperature changes in the two samples.

**Figure 6:**
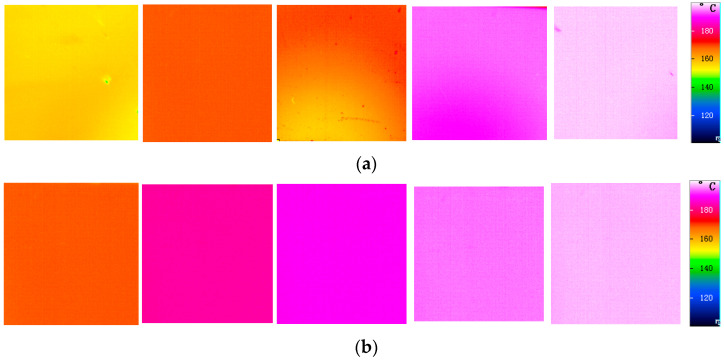
The infrared distribution maps of the two samples. (**a**) Si; (**b**) Diamond film.

Figure 6a shows the infrared distribution map of the Si film sample during the heating process to 200 °C. Due to the poor thermal conductivity of the material, local bright spots will appear on the sample, indicating an uneven heat dissipation phenomenon. Figure 6b shows the heat dissipation effect of the diamond film when heated to 200 °C. The infrared image indicates that there are no concentrated bright spots on the surface of the diamond film, and the heat is evenly distributed. This is because the diamond film has a strong heat conductivity.

### 3.5. The Heat Dissipation Effect of Diamond Chips

In order to study the thermal management capability of the samples under actual application conditions, diamond film is placed on the surface of the GaN power amplifier chip. The chip is placed on a cooling plate. The heat dissipation effects of the two devices under a heat source power of 2 W/cm^2^ are shown in Figure 6.

**Figure 7:**
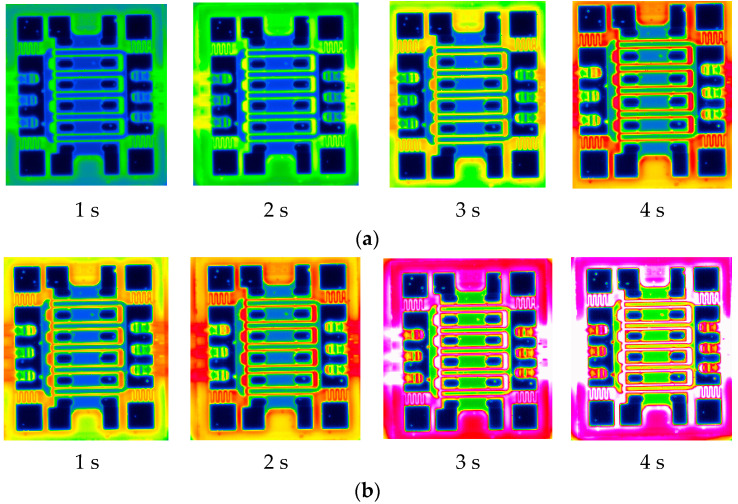
The infrared distribution maps of the Si and diamond film on a power amplifier. (**a**) Diamond film. (**b**) Without diamond film.

Figure 7a shows the infrared distribution maps of the Si substrate and the diamond film on the power amplifier. The infrared image indicates that the power amplifier is significantly influenced by the diamond film. The junction temperature of the power amplifier is limited to a maximum of 35 °C. The chip exhibits uniform heat dissipation with no localized bright spots.

In contrast, as shown in Figure 7b, in the absence of the diamond film, the maximum junction temperature of the power device reaches 65 °C, and bright spots appear on the device as the heating time increases.

**Figure 8:**
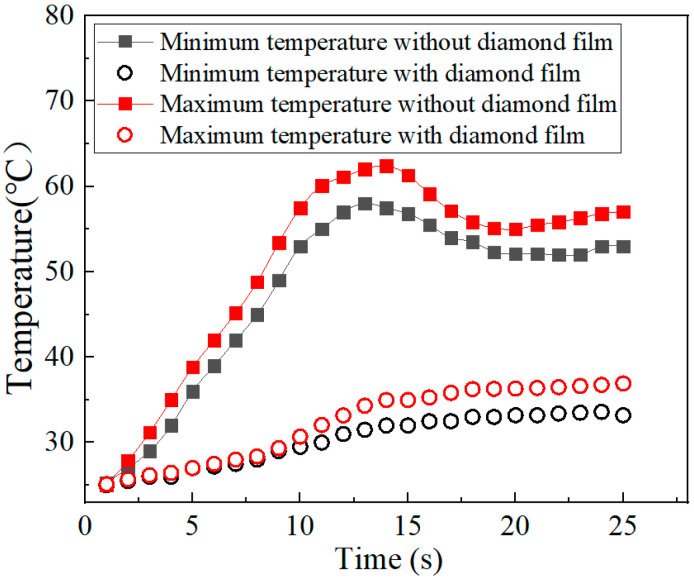
The temperature variations in the power amplifier chip with or without diamond film.

Figure 8 shows that when the diamond film is absent, the temperature of the power amplifier rises rapidly to 65 °C. Then, under the effect of the heat sink, the junction temperature decreases, with the maximum junction temperature remaining at 58 °C and the minimum temperature maintaining at 54 °C. When the diamond film is present, the maximum temperature of the power amplifier chip shows a slow increase and then stabilizes. The maximum temperature remains at 35 °C, while the minimum temperature stays at 30 °C. The results indicate that the power amplifier chip has a better heat dissipation effect under the action of the diamond film. Compared with the case without the diamond film, the temperature of the power amplifier chip drops significantly by approximately 30 °C.

## 4. Conclusions

Diamond films possess unparalleled thermal properties and extremely excellent optical properties, which enables them to solve many bottlenecks in high-end technical fields. The high and stable thermal conductivity and optical properties are key indicators for advanced power device packaging materials. It ensures the measurability and controllability of junction temperature. However, high-quality CVD diamond films have slow growth rates, high energy consumption, and expensive equipment, resulting in high costs and making it difficult for large-scale commercial application. Therefore, this project proposes a DC Arc Plasma Jet CVD technology, which is a method for preparing polycrystalline CVD diamond films on titanium-coated graphite substrates. The scanning electron microscope results show that the diamond films prepared by this method have the characteristics of intact crystal structure and very few defects, and can achieve large-area and uniform epitaxial growth, as well as high surface flatness. The XRD results show that the structure of the diamond film is extremely stable at high temperatures. The Raman results show that the position and shape of the diamond characteristic peaks do not change with temperature, and the TG results indicate that the weight loss of the diamond film during heating remains at an extremely low level of 0.1 mW/mg. The DSC curve is smooth, with no obvious endothermic or exothermic peaks. All of these indicate that the diamond film has a stable crystal structure and stable thermal properties. In the mid-infrared spectral region at 3.3–3.6 μm (2780–3030) cm^−1^, there exists C-H stretching vibration absorption. When the absorption coefficient at 2830 cm^−1^ is greater than 5.5 cm^−1^, there is a good linear relationship between thermal conductivity and absorption coefficient. When the absorption coefficient at 2830 cm^−1^ is less than 5.5 cm^−1^, the influence of infrared transmittance is not significant. Therefore, within a certain range, infrared transmittance can be used as a rapid method to determine the thermal conductivity of diamond films. The infrared results show that the diamond film has excellent thermal diffusion capability, ensuring uniform heat distribution and eliminating the fatal hotspots. This reduces the junction temperature of the power device chip from 65 °C to 35 °C, indicating a significant improvement in the reliability and service life of the device.

The DC arc plasma jet CVD technology proposed in this paper is expected to break through the cost and scale barriers of high-performance diamond films, transforming them from a “luxury item” in the laboratory to a “key material” available in the high-end industrial field. This will provide strong material support for revolutionary progress in fields such as next-generation high-power electronic devices and optoelectronic devices.

## Figures and Tables

**Table 1:** The main deposition parameters of diamond films.

Temperature/°C	Power/kW	Pc/kPa	Duration/h	CH_4_ (sccm)	H_2_ (sccm)	N_2_ (sccm)	Ar (sccm)
850~900	15	12	200	120	20~40	0~50	10~30

## Data Availability

The original contributions presented in this study are included in the article. Further inquiries can be directed to the corresponding author.
